# Encapsulation of Thymol in Ethyl Cellulose-Based Microspheres and Evaluation of Its Sustained Release for Food Applications

**DOI:** 10.3390/polym16233396

**Published:** 2024-12-02

**Authors:** Iro Giotopoulou, Haralambos Stamatis, Nektaria-Marianthi Barkoula

**Affiliations:** 1 Department of Materials Science and Engineering, University of Ioannina, GR-45110 Ioannina, Greece; i.giotopoulou@uoi.gr; 2 Department of Biological Applications and Technology, University of Ioannina, GR-45110 Ioannina, Greece; hstamati@uoi.gr

**Keywords:** thymol, entrapment, microparticles, microspheres, ethyl cellulose, active packaging

## Abstract

Food waste is a global concern with enormous economic, environmental and social impacts that has contributed to active packaging evolution. However, incorporating bioactive substances into the packaging can deteriorate its physicochemical and mechanical characteristics. Thus, the objective of this work was to entrap the natural bioactive compound thymol into microparticles and apply them in the form of pads for the controlled release of bioactivity in food packaging material. The physicochemical characteristics and bioactivity of five different ethyl cellulose-based microparticles were evaluated. Increasing the amount of thymol in the formulation led to higher encapsulation efficiency. Encapsulation resulted in a substantial increase of >10–20 °C in the volatilization temperature of thymol, and the release of thymol occurred following a sustained profile, best described by the Higuchi release kinetic model. Increasing the polymer to thymol ratio in the microparticles resulted in higher thermal stability and a more gradual release profile. While all formulations demonstrated considerable inhibition of *E. coli* growth, the ones with the highest thymol content maintained their antimicrobial activity for at least one month of microparticle storage. Furthermore, the ability of the microparticles in retaining pH and titratable acidity of cherry tomatoes was evaluated, and it was confirmed that these characteristics were maintained during 21 days of storage.

## 1. Introduction

Annually, many tons of food are wasted, leading to huge economic losses and resource wasting, in addition to water and air pollution from the decomposition of the food scraps [1,2,3]. Recent data on food waste [4] indicate that conventional methods for food preservation are not adequate, while chemical preservatives do not meet consumers’ perspectives. Therefore, the food industry seeks innovative and sustainable methodologies to increase the shelf-life of food products. Since microbial activity is a major reason for food spoilage and food-borne illnesses, one promising innovation in this field is to facilitate food preservation via the use of antimicrobial active packaging. In this context, great endeavors have been made to utilize natural bioactive compounds in food preservation. Essential oils are natural bioactive substances derived from plants, with a wide range of antimicrobial and antifungal properties, and they are generally recognized as safe (GRAS) [5,6,7,8]. Nevertheless, their strong aroma and volatile nature, in addition to the suppression of their antimicrobial activity in case of direct contact with complicated food systems [9] (e.g., proteins, lipids), restrain their direct application for food preservation.

To overcome these challenges, various methods have been employed to integrate bioactive substances into packaging. However, the incorporation of bioactive substances into packaging material may deteriorate its mechanical properties, which can eventually result in exposing the food to harmful environmental conditions [10,11,12,13,14]. Thus, a promising simple and cost-effective solution is to entrap bioactive substances into various materials and place them separately into the packaging in the form of sachets, pads or capsules [15]. These methods have been commercially employed as an efficient approach for the incorporation of oxygen absorbers, such as ferrous iron oxides [16,17,18], into packaging to control oxygen concentration. Furthermore, volatile or nonvolatile active substances, including essential oils [19,20,21,22], ethanol [23], precursors for chlorine dioxide production [24], catechol, ascorbic acid and oxidative enzymes [23], have also been incorporated into packaging in the form of sachets [17,25]. The fabrication process involves entrapment of active substances into carriers through adsorption, absorption or grafting followed by enclosing these carriers into permeable sachets. A plethora of materials have been utilized as carriers and sachets, including β-cyclodextrin [19], activated carbon or zeolites [23], low-density polyethylene [20], nonwoven fabric, fiber cellulose tea bags [19], paper laminate with ethylene vinyl acetate and porous polypropylene [17,21]. The complexity regarding the multiple materials involved in this three-step procedure could be overcome by the employment of encapsulation methodologies.

Encapsulation is a powerful methodology that entraps active substances into a polymeric matrix, resulting in either particle formation when active substances are dispersed in the matrix or capsules when active substances are surrounded by the polymeric matrix, which is usually referred to as a wall [26,27]. Additionally, encapsulation can result in complexes when active substances are spatially deposited into an open source. A wide range of encapsulating techniques have been utilized for the encapsulation of essential oils or phenolic compounds, including spray drying [28,29,30,31,32,33,34,35,36], freeze drying [34,37], coacervation [38], inclusion complexation [15], liposome entrapment, ionic gelation [39,40,41], electrospray and solvent evaporation. Among those diverse methodologies, spray drying is more suitable for heat-stable active substances [26], freeze drying requires long processing times, ionic gelation and inclusion complexation have been reported to possess limited loading capacity [40], and the coacervation technique requires toxic cross-linking agents [42], whereas entrapment into Liposomes is restrained by economic factors. Opposingly, particle formation by solvent evaporation is a feasible method capable of entrapping both hydrophilic and hydrophobic active substances at ambient conditions [43], without requiring specialized equipment. The methodology frequently comprises a dissolution step of the polymer along with the active ingredient into an organic volatile solvent such as dichloromethane, chloroform or ethyl acetate, followed by mixing with an aqueous phase in the presence of an emulsifier to form an emulsion. By applying heat or by reducing pressure, the volatile solvent evaporates and the polymer forms microparticles in the shape of spheres [44,45,46].

Proper selection of the polymeric materials can result in a sustained and controlled release of active substances extending their effectiveness over time, whereas an improper selection of the enclosure materials could result in a burst loss of bioactive substances or restrain their release and subsequently their availability. Ethyl cellulose is a nontoxic cellulose derivative often employed in pharmaceuticals for controlled release due to its hydrophobic and non-swellable nature [47,48]. Although ethyl cellulose has been studied for the encapsulation of various drugs through the solvent evaporation method [49,50,51,52,53], its employment in active packaging applications has not been yet evaluated.

Within this context, the objective of this study was to fabricate ethyl cellulose microparticles with entrapped thymol using a green method for food packaging applications. The relation between the different microparticle formulations and their physical, chemical and bioactive characteristics was assessed. Particularly, the amount of thymol entrapped, the antimicrobial activity and the release mechanism were evaluated. Finally, the microparticles were incorporated into the packaging of cherry tomatoes in the form of a patch to evaluate the microparticles’ efficiency in maintaining the quality of cherry tomatoes during storage.

## 2. Materials and Methods

### 2.1. Materials

Thymol (2-isopropyl-5-methylphenol, purity ≥99%), ethyl cellulose (EC) 100 cp, n-Hexane (≥98%), Sodium hydroxide, Span 80 (Sorbitan oleate) and Whatman qualitative filter paper (Cat No 1004055, pore diameter 20–25 μm) were purchased from Sigma Aldrich (Steinheim, Germany). Methanol (Purity ≥ 99.8%) was purchased from Honeywell Fluka (Seelze, Germany). Paraffin oil was a pharmacy product under the trademark Nujol^®^ (Produced by Famar for Bayer, Athens, Greece).

### 2.2. Methods

Thymol microparticles were fabricated according to the methods proposed by previous studies [54,55,56], with slight modifications. Briefly, 5% (*w*/*v*) or 10% (*w*/*v*) of ethyl cellulose and thymol were dissolved in the solvent (1:9 (*v*/*v*) methanol/acetone). After mixing, the polymer/thymol mixture was emulsified into paraffin oil with a 1% (*v*/*v*) or 2% (*v*/*v*) Span 80 under continuous stirring at 400rpm. The mixing ratio between the two liquid phases was 1:5 (*v*/*v*). The solvent evaporated during overnight mixing, and the microparticles were washed with n-hexane, filtered and naturally dried. This resulted in solid particles, without the need to apply any lyophilization steps. The solid particles were subsequently characterized with the methodologies described in the following paragraphs (i.e., Section 2.2.1, Section 2.2.2, Section 2.2.3, Section 2.2.4, Section 2.2.5, Section 2.2.6 and Section 2.2.7). An overview of the used formulations and the designation of the prepared microparticles is included in Table 1. The microparticles were weighed, and the recovery was calculated using the following formula (Equation (1)):

(1) $$Recovery\left(\%\right)=\frac{Weight\;of\;the\;microparticles\;obtained\;(g)}{Theoretical\;weight\;(g)}\times 100$$

#### 2.2.1. Microscopy

To examine their morphology, the microparticles were placed on a glass slide on the stage of an optical microscope under 4× magnification lenses. Images were taken with the help of a camera (Bresser MicroCamLab, Bresser GmbH, Rhede, Germany), and the sizes of 20 microparticles of each batch were measured with a Bresser MikroCamLabII © 2003–2009 V3.7. 

#### 2.2.2. ATR-FT-IR

An Agilent 4300 handheld FTIR spectrometer (Agilent Technologies, Inc., Santa Clara, CA, USA) was used to collect the Fourier transform infrared spectra of the microparticles with and without thymol, with an external device for attenuated reflectance (ATR). Specifically, before any measurement, a background spectrum from the empty cell was received, and for the sample measurements, one hundred eighty-four scans were performed with a resolution of 4 cm^−1^ in the wavenumber range of 600–4000 cm^−1^. To eliminate baseline drift, correction of the raw ATR-FTIR data was performed with the aid of the Savitsky–Golay smoothing technique, followed by vector normalization to correct sample deviations, such as concentration or thickness. Integration of the characteristic peaks of thymol and Span 80 were performed by the sum of absolute trapezoid values (Integrate gadget in OriginPro 9.0, 2013).

#### 2.2.3. Simultaneous Thermal Analysis (STA)

STA was performed using a Netzsch-STA 449C Jupiter instrument, (Netzsch GmbH, Selb, Germany). A quantity of 7–10 mg of microparticles with or without thymol were heated under a 40 mL/min nitrogen flow from 20 °C to 610 °C at a heating rate of 20 °C/min. This enabled thermogravimetric measurements (TGAs) to be performed simultaneously with differential scanning calorimetry (DSC) ones. The loading/content was estimated from the TGA thermographs of the dried microparticles with and without thymol, and based on these results, the encapsulation efficiency was defined. More specifically, based on the mass loss data assigned to thymol degradation, the loading capacity (LC) and the respective encapsulation efficiency (EE) of the prepared microparticles were calculated according to Equations (2) and (3) [57].

(2) $$LC\%=\frac{weight\;of\;encapsulated\;thymol}{weight\;of\;microparticles}\%$$

(3) $$EE\%=\frac{weight\;of\;encapsulated\;thymol}{weight\;of\;thymol\;added\;in\;the\;formulation}\%$$

#### 2.2.4. Antimicrobial Activity

The antibacterial activity was tested against gram (−) *Escherichia coli* BL21DE3, as described before [58,59]. The strains used were from our lab collection and the Health Protection Agency, Porton Down, Salisbury, UK. Fresh bacterial culture was grown in sterile Lysogeny Broth (LB). A bacterial population from the exponential phase was added to 0.9% *w*/*v* NaCl solution (~10^7^ CFU mL^−1^). Different amounts of microcapsules (app. 5 mg and 10 mg) from each formulation were added into Eppendorf tubes containing 100 μL of the above bacterial population and incubated for 48 h in a cold chamber. A quantity of 100 μL of bacteria in the absence of the sample were used as a control. Then, 25 μL of the bacterial population that interacted with the sample were inoculated into 225 μL of fresh sterile LB medium in a 96-well Elisa microplate. The microplate was then incubated at 37 °C under stirring, and measurements of the O.D. at 600 nm were taken every hour for eight hours. The antimicrobial activity of the microparticles was evaluated as a function of time during storage in sealed conditions at an ambient temperature of 25 °C ± 2. All measurements were performed in triplicate.

#### 2.2.5. Release of Thymol

The release of thymol from the developed microparticles was assessed using 10% *v*/*v* ethanol medium, which is assigned to foods with a hydrophilic character [60]. In detail, 0.01 g of each microparticle formulation was immersed into 2 mL of the liquid food simulant and remained at 25 °C without agitation. Thymol release was examined over 30 days (i.e., 720 h) since this duration aligns with the timeframe reported in the literature at which tomatoes could be stored [61,62,63,64]. At predetermined time intervals, 200 μL of the liquid medium were transferred to an Elisa plate, and with the aid of the Elisa microplate spectrophotometer (Thermo Fisher Multiskan SkyHigh, Thermo Fischer Scientific, Bremen, Germany), a thymol characteristic UV absorption peak at 273 nm was detected, and the concentration of thymol was calculated with the aid of the calibration curve.

#### 2.2.6. Tomato Storage (pH and Titratable Acidity)

pH and titratable acidity of tomatoes stored both with thymol microparticles and without were calculated according to the methodology proposed by Loro et al. [65] and Gawad Saad et al. [66], with slight modifications. Fresh cherry tomatoes were selected based on uniform color and the absence of any visible injuries, bruises or signs of spoilage and washed thoroughly with detergent and water. Tomatoes were separated into batches weighing 100 g and placed into a polyethylene terephthalate perforated rigid container in which a tape with 0.5 g of microcapsules was attached, whereas a batch of 100 g was placed into an identical rigid container without any active substances to serve as the control. The perforations in the packaging walls facilitate air circulation, support fruit respiration and prevent moisture buildup. The storage temperature was 25 °C to simulate the supermarket’s storage conditions. At predetermined time intervals (0th day, 7th day, 14th day and 21st day), pH titratable acidity and weight loss were measured. To determine the pH, two to three tomatoes were blended, 1 mL of homogenized tomato juice was diluted in 5 mL of distilled water and pH reading was performed by direct immersion of the digital pH meter electrode after its calibration in buffer solutions. Total titratable acidity was calculated after titration of the diluted juice with 0.1N NaOH until a pH of 8.1. The volume of NaOH consumed was used to calculate the g of citric acid per 100 g sample (%TA) [65,66]. The measurements were performed in two to three randomly selected cherry tomatoes.

#### 2.2.7. Statistical Analysis

All analyses were carried out in triplicate, in which the results were recorded as mean ± standard deviation. Student’s *t*-test, one-way ANOVA analysis and Tukey’s multiple comparison test were implemented using IBM SPSS Statistics version 21 (SPSS Inc., Chicago, IL, USA). *p*-value is a measure of the likelihood that any observed difference between groups occurred by chance. A *p*-value less than or equal to 0.05 is considered statistically significant, indicating a meaningful difference. A *p*-value less than or equal to 0.01 indicates a very significant statistical difference, while *p*-values less than 0.001 denote highly significant statistical differences.

## 3. Results and Discussion

### 3.1. Physicochemical Characterisation

#### 3.1.1. Recovery and Mean Particle Size

**Figure 1:**
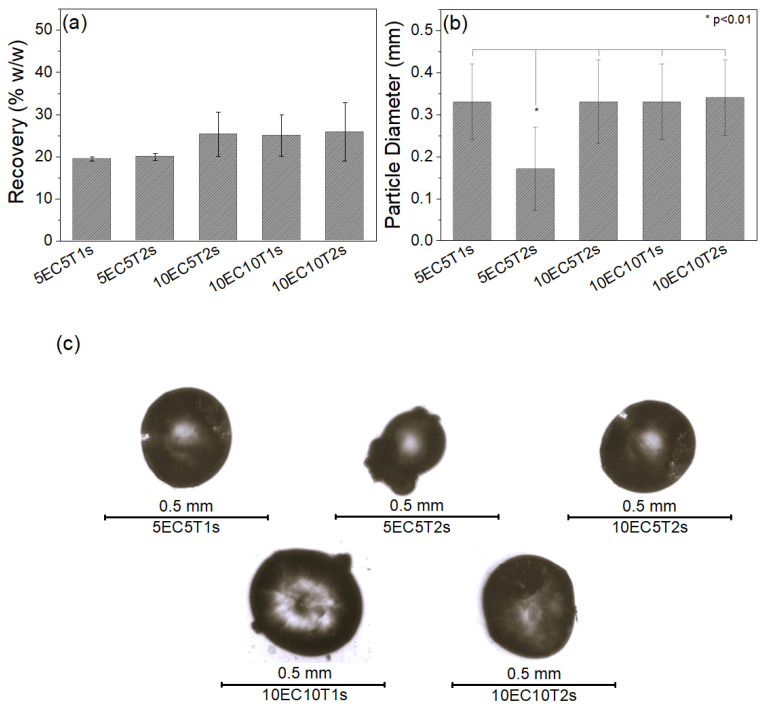
Microparticle mass percentage recovery (**a**), particle diameter (**b**) and optical microscopy images of microparticles of each microparticle formulation (**c**). * *p* < 0.01.

Recovery (%) of the microparticle’s different formulations appears in Figure 1a. A slight increase of % recovery is observed for the formulations containing a higher amount of polymer (10% *w*/*v* vs. 5%), without, however, any significant difference in the recovery among the different formulations with 10% *w*/*v* ethyl cellulose. The % recovery is relatively low since a considerable amount of material was aggregated and disregarded during the filtration process. The recovery capability of the proposed methodology could be enhanced by increasing the volume of paraffin oil and consequently providing the droplets with more volume to move freely and avoid aggregation. Furthermore, increasing the volume of the medium has been reported to result in smaller particle sizes due to the same reason [49]. Optimization of system’s stirring speed was performed before preparing the microparticles since it strongly affects their size [53,57], and finally, all the samples were prepared with the same optimum speed. As observed in Figure 1b, most of the formulations resulted in almost identical particle diameters, with the only exception being the 5EC5T2s formulation, which presented the smallest mean particle diameter. This variation was attributed to the lower amount of polymeric phase, in conjunction with the higher surfactant content. Surfactants not only stabilize the droplets and prevent aggregation but also decrease interfacial tension between the two phases, facilitating droplet fragmentation during emulsification [67]. Increasing polymer concentration has been reported to possibly cause turbulence, which is followed by diverse particle sizes and larger particle size distributions [57]. Furthermore, increasing polymer concentration increases the viscosity, and consequently, larger droplets are formed [53]. There was no significant difference to the mean particle diameter between the other particle formulations. Similar sizes have been reported in the literature [49,53,68].

The optical microscopy images of the various microparticle formulations are depicted in Figure 1c. Please note that a scale bar corresponding to 0.5 mm has been included in the respective images presented in Figure 1c. The microparticles appear spherical, with a homogenous surface without any visible pores. Aggregates of some spheres are also depicted in Figure 1c. To surpass aggregation, a potential solution would be to increase the amount of paraffin oil since this would provide more space for the microparticles to move freely.

#### 3.1.2. FTIR-ATR

**Figure 2:**
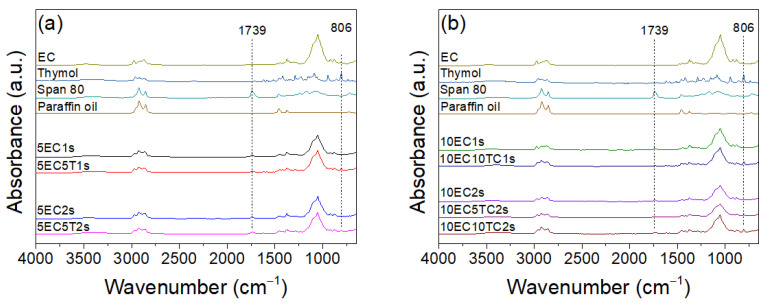
FTIR-ATR spectra of ethyl cellulose thymol, Span 80 and the respective formulations without (**a**) and with (**b**) thymol.

The FTIR-ATR spectra of reference formulations (i.e., without thymol) are presented in Figure 2a. The ethyl cellulose spectrum depicts a broad absorption peak at 3492 cm^−1^ due to –OH stretching vibrations on ethyl cellulose chains. The peaks at 2974 cm^−1^, 2926 cm^−1^ and 2873 cm^−1^ are assigned to aliphatic C-H stretching vibrations; the high intensity peak at 1051 cm^−1^ denotes the stretching vibration of C-O-C bonds, which is characteristic of the β (1→4) glycosidic bonds or ethoxy groups in the cellulose ring; the weak intensity peaks at 1374 cm^−1^ and 1355 cm^−1^ are ascribed to C-H bending; and O-H bending while peaking at 882 cm^−1^ may be associated with C-H out of plane bending from the ethoxy groups [57,69]. The absorption peak of Span 80 emerging in 3423 cm^−1^ is associated with O-H stretching vibrations; the peak at 1739 cm^−1^ is ascribed to the C=O stretching vibration; and C-H bending is associated with the absorption peak at 1467 cm^−1^, while absorption peaks at 1169 and 1083 are due to C-O stretching vibrations 721 cm^−1^ [70]. As anticipated, paraffin oil exhibits prominent absorption peaks at 2919 cm^−1^, 2855 cm^−1^ and 1457 cm^−1^, corresponding to C-H stretching vibrations, along with a medium intensity peak at 1377 cm^−1^ attributed to C-H bending vibrations [70]. The two low-intensity peaks of the thymol spectrum at 1618 cm^−1^ and 1585 cm^−1^ are assigned to C=C stretching vibrations, which is typical for conjugated double bonds; the peaks emerging at 1457 cm^−1^, 1379 cm^−1^ and 1361 cm^−1^ could be associated with methyl C-H in plane bending, scissoring and deformation, while peaks emerging at 1419 cm^−1^ and 1343 cm^−1^ could be due to in-plane bending of O-H groups attached to the aromatic ring. C-O stretching vibrations in the aromatic ring are associated with peaks at 1228 cm^−1^ and 1152 cm^−1^, respectively, while C-H of the aromatic ring in-plane bending vibrations could be associated with the peak at 1088 cm^−1^, and aromatic C-H out-of-plane bending could be associated with the peaks at 945 cm^−1^ and 806 cm^−1^ [71,72].

In the spectra of all various microparticle formulations containing thymol, illustrated in Figure 2b, a new peak emerges at 806 cm^−1^, which can be attributed to the aromatic C-H out-of-plane bending of thymol. Furthermore, the O-H stretching peak broadens and shifts towards smaller wavenumbers, indicating the formation of intermolecular hydrogen bonds. An additional noteworthy observation is the presence of the peak at 1739 cm^−1^, which is associated with Span 80 and is observed in all microparticles with or without thymol. The above ATR-FTIR analysis confirmed that both thymol and Span 80 were deposited in microparticles. To shed light on the microparticles’ composition, a peak analysis was conducted. According to the data derived from the analysis, the ratio between Span 80 and thymol was calculated, and the corresponding results are shown in Table 2. 5EC5T1s and 10EC10T1s exhibited the lower Span 80 to thymol ratio compared to other microparticle formulations, including 5EC5T2s, 10EC5T2s and 10EC10T2s. Thus, increasing the amount of Span 80 during the fabrication stage resulted in microparticles with higher surfactant content. Although there was no significant difference among the samples prepared with a higher amount of Span 80, formulation 5EC5T2s seem to have the highest surfactant content when compared to the other two formulations (10EC5T2s and 10EC10T2s), possibly due to the lower amount of polymer used at the fabrication stage.

#### 3.1.3. STA Analysis

**Figure 3:**
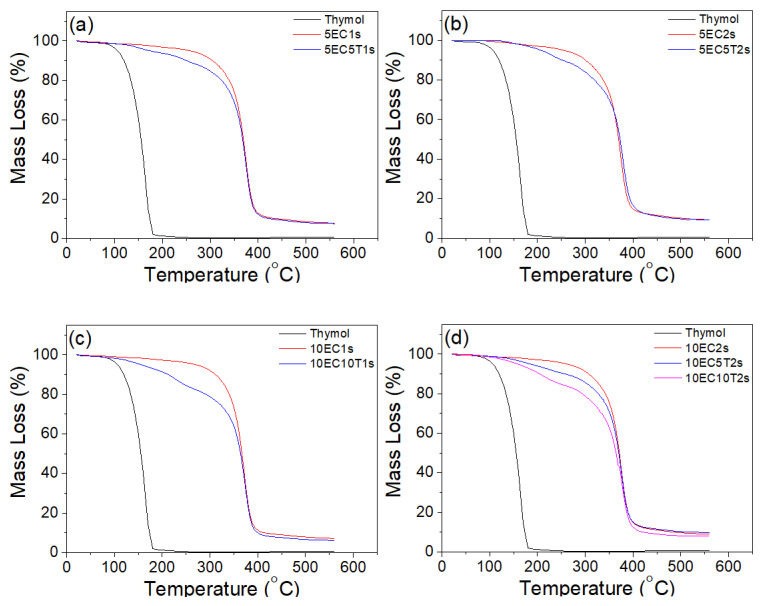
Thermographs of thymol and microparticle formulations both with and without thymol: formulations with 5EC and 1s (**a**), formulations with 5EC and 2s (**b**), formulations with 10EC and 1s (**c**), formulations with 10EC and 2s (**d**). Thymol as reference has been added in all graphs.

STA was used to assess the thermal properties of the thymol microparticles and determine the LC and EC of the prepared microparticles. Based on the mass loss spectra (TGA) presented in Figure 3 and the respective DTG data (Figure S1), thymol’s thermal degradation occurs at a single step, which ends at approx. 200 °C [73]. Similarly, ethyl cellulose-based microparticles without thymol present a single step degradation that starts at around 250 °C (onset temperature) and ends at about 500 °C [74]. The thermal degradation of all thymol-loaded microparticles follows a multi-step pattern that is better depicted in the DTG curves (Figure S1). The initial two steps were assigned to thymol loss. The first step (up to approx. 200 °C) designates the amount of thymol that is easily released from the capsules, while the second one (between approx. 200 and 250 °C) illustrates the amount of thymol that is less mobile due to the formation of intermolecular hydrogen bonds between thymol and ethyl cellulose, in accordance with the FTIR-ATR data. Finally, the last step was related to ethyl cellulose thermal degradation.

The TGA data are further supported by the DSC data (Figure S2). Two endothermic peaks are depicted in the DSC curves of thymol at 55 °C and 171 °C due to melt and degradation, respectively. These two peaks are almost absent from the respective curves of thymol-based microparticles. As discussed previously, the total or partial disappearance of thermal events is generally taken as a proof of complex formation [75]. Microparticles without thymol present an endothermic peak at ∼194 °C, which is associated with the solid phase–mesophase transition [76]. However, this peak is less distinct in the DSC graphs of the microparticles with thymol, which can be assigned to interactions between thymol and ethyl cellulose. A possible explanation could be the plasticizing effect of thymol, which reduces the intermolecular interactions of ethyl cellulose chains, resulting in an increased molecular mobility and a less distinct DSC peak. Additionally, molecular interactions such as hydrogen bonds between thymol and ethyl cellulose could result in higher energy requirements for the transition to occur, which eventually could modify the characteristic peak of the transition.

To assess thymol microparticles’ thermal stability, the temperature at which 20% thymol loss occurs was calculated, and the results are depicted in Table 3. Thymol loses 20% at 130 °C while 20% loss of thymol from the microparticles occurs at 140 °C, indicating that incorporation of thymol into ethyl cellulose microparticles increased thymol’s thermal stability. Increasing the ethyl cellulose to thymol ratio in the formulation causes an increase of thymol’s thermal stability at 150 °C. Upon further analysis of the thermograms, thymol content (i.e., LC) within the microparticles was identified, and the corresponding results are included in Table 3. 10EC10T1s entrapped the highest amount of thymol, followed by 10EC10T2s. Thymol’s content in formulation 5EC5T1s was slightly higher than 5EC5T2s and 10EC5T1s despite that the loading of thymol was the same. This can be related to the higher amount of Span 80 in these formulations, which resulted in decreasing thymol entrapment. Based on the LC data, the EE was calculated, and as reported in Table 3, it varied between approx. 11 and 26.5%. This quite low encapsulation performance of the prepared microcapsules does not fully reflect the ability of EC to entrap the active agent thymol. Rather, it could be linked with the very low recovery efficiency of the processes, which resulted in high amounts of aggregates. These aggregates have been disregarded during the filtration stage. The actual amount of thymol that was available to be encapsulated could not therefore have been appropriately assessed since the relative content of thymol and ethyl cellulose in the aggregates was not estimated.

#### 3.1.4. Thymol Release

**Figure 4:**
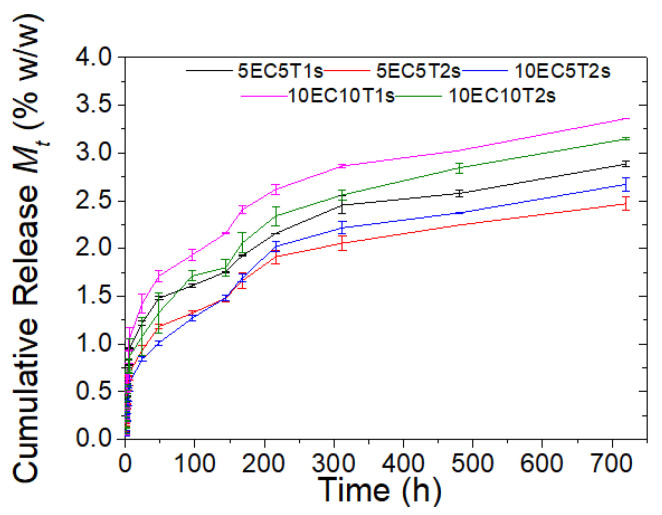
Cumulative release of thymol into a liquid that simulates hydrophilic foods as a function of time and the microparticle’s formulation.

Based on the results presented in Figure 4, thymol’s release from microparticles presents a biphasic profile, with an initial burst followed by sustained release since the slope of the release diagram versus time changes at 150 h. This interesting phenomenon may be attributed to the diverse release rate of thymol present on the microparticle’s surface and that is entrapped inside the microparticles. At this initial phase, 1.5–2% *w*/*w* of thymol is released from microparticles towards the food simulant. It has been reported that release in EC-based microparticles occurs in three stages, including an initial rapid diffusion from the microparticle’s surface, followed by slower release due to polymer hydrolysis and a final rapid release, which is attributed to the erosion of the polymeric capsule [50].

In line with the literature observations, the release rate was influenced by the bioactive substance to polymer ratio [77] as well as the surfactant content. The formulation with the highest amount of thymol and the minimum amount of surfactant, i.e., 10EC10T1s, presented the highest cumulative release after 700 h, followed by 10EC10T2s. Similarly, cumulative release of thymol from 5EC5T1s was higher than that of the 5EC5T2s microparticles. This, in conjunction with the TGA results, suggests that the higher amount of surfactants results in lower thymol content, and in turn lower released bioactivity. Comparing the microparticles’ release during the initial phase, it can be concluded that the formulation 10EC5T2s releases less thymol compared to the formulation 5EC5T2s, despite having less thymol content (observed from TGA analysis), possibly due to the higher hydrophobic polymer content, which suppresses the release. It is noteworthy that at the initial burst release phase, the 5EC5T1s microparticles release a higher amount of thymol despite having lower thymol content than 10EC10T2s. This finding could be assigned to the lower polymer and/or Span 80 content of the formulation 5EC5T1s since their hydrophobic nature could inhibit thymol’s release towards the aqueous solution. Overall, it can be concluded that all the different formulations presented a gradual sustained release. Furthermore, through the adjustment of the composition, it is possible to control the release rate and profile of the bioactivity, supporting the different needs of the packaging industry.

The release kinetics was evaluated by fitting the data by kinetic models [78,79] often used to study the release of active ingredients from microparticle samples [77,80,81,82,83,84,85]. These models are zero order (Equation (4)), first order (Equation (5)), Hixson–Crowell (Equation (6)), Higuchi (Equation (7)), and Korsmeyer–Peppas (Equation (8)), as described by the respective equations.

(4) $$M_{t}=M_{0}+K_{0}t$$

(5) $$\ln\;M_{t}=\ln\;M_{0}-K_{1}t$$

(6) $${M_{0}}^{1/3}-{M_{t}}^{1/3}=K_{HC}t$$

(7) $$M_{t}=K_{H}\sqrt{t}$$

(8) $$M_{t}=M_{\infty }K_{KP}t^{n}$$

M*_t_* is the cumulative percentage bioactive substance release at time *t*; M_0_ is the amount initially released (at *t* = 0). K is the rate constant of each kinetic model, followed by an index of 0, 1, HC, H or KP for zero order, first order, Hixson–Crowell, Higuchi and Korsmeyer–Peppas kinetic models, respectively.

Based on the kinetic modeling results presented in Table 4, all microparticles are best described by the Higuchi release model, with R^2^ values exceeding 0.9, as corroborated by findings from other authors [50,52,78]. This suggests that diffusion is the main mechanism behind the release of the bioactivity, while matrix swelling and dissolution are negligible [78]. Furthermore, matrix erosion does not seem to affect thymol’s release since the data do not fit well to the Hixson–Crowell model, with R^2^ values at about 0.5. A first-order model is used to describe the dissolution of an active substance, including the dissolution of water-soluble substances [79]; therefore, as expected, the data fit poorly to this model. A notable observation is the relatively good fit to the zero-order release model (with R^2^ ∼ 0.7), which indicates release at a constant sustained rate, therefore retaining the active substance’s concentration for an extended period [79,86,87]. Formulation 10EC5T2s demonstrates a higher correspondence to the zero-order model, with an R^2^ value of 0.8 when compared to the rest formulations, manifesting that by increasing the polymer to thymol ratio, a zero-order release could be accomplished. Although the Korsmeyer–Peppas kinetic release model has been reported as unsuitable for describing release from swellable polydisperse matrices, the high R^2^ values indicate that we can still evaluate the “n” value. The “n” values of all the microparticles except 10EC5T2s are below 0.43, which indicates that the transport mechanism follows Fickian diffusion, while the “n” value of the formulation 10EC5T2s is greater than 0.43 and smaller than 0.85, which signifies an anomalous transport in which diffusion and swelling coincide.

### 3.2. Bioactivity

#### 3.2.1. Antimicrobial Activity

**Figure 5:**
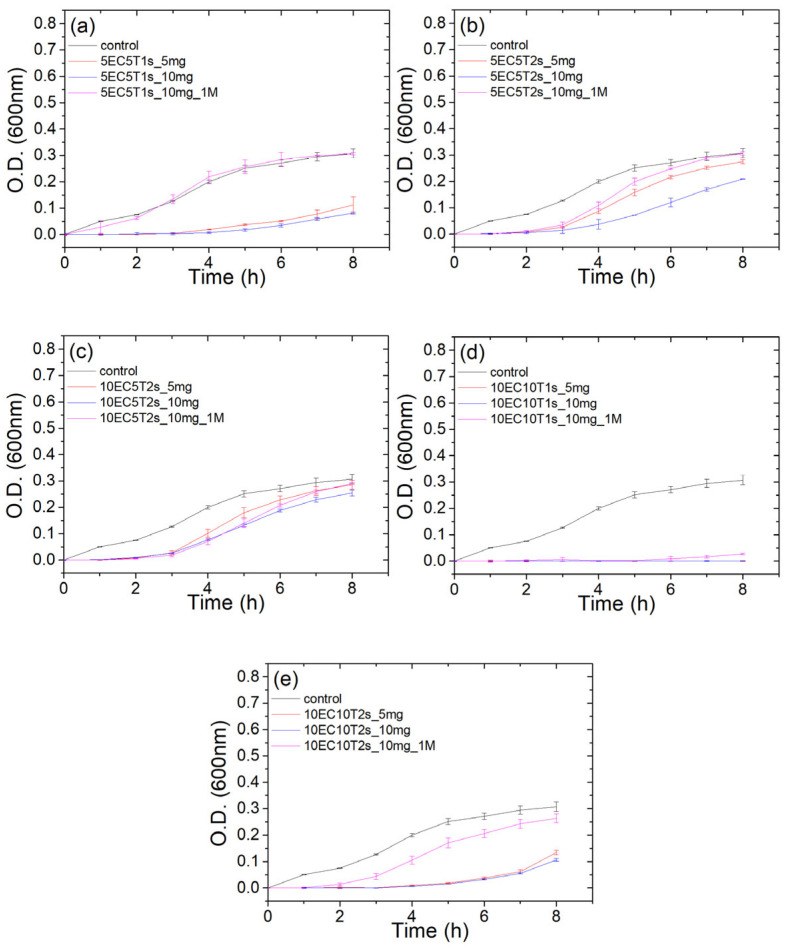
Antimicrobial activity against *E. coli* for varying amounts of microparticles (5 mg, 10 mg) both initially and after one month of storage (_1M). The formulations tested were 5EC5T1s (**a**), 5EC5T2s (**b**), 10EC5T2s (**c**), 10EC10T1s (**d**) and 10EC10T2s (**e**). The effect of particle storage in ambient conditions was also assessed.

The bioactivity of microparticles was assessed against *E. coli* as a gram (−) representative bacterium, which is a common food contaminant. Microparticle bioactivity after one month of storage at ambient conditions was also evaluated. As observed in Figure 5, microparticles with formulation 5EC5T1s fully inhibited *E. coli* growth; nevertheless, after one month of storage, their antimicrobial activity diminished. Microparticles with the formulations 5EC5T2s and 10EC5T2s exhibited the smallest inhibition of *E. coli* growth. This finding can be associated with the higher Span 80 loading in formulation 5EC5T2s and the higher polymer to thymol ratio in formulation 10EC10T2s since Span 80 and ethyl cellulose are both highly hydrophobic, and they can retard thymol’s release towards the aqueous medium. Microparticles with formulation 10EC10T1s fully inhibited *E. coli* growth, and the antimicrobial activity was retained after one month of storage at ambient conditions. Similarly, 10EC10T2s presented almost full inhibition of *E. coli* growth, and after one month of storage inhibited *E. coli* growth, although its antimicrobial activity decreased. Overall, after one month of storage, formulations with the lowest thymol content lost their antimicrobial activity. Ethyl cellulose-based microspheres have demonstrated up to 12-week stability [88]; thus, the potential of the 10EC10T1s microparticles to maintain their stability for longer periods is high.

#### 3.2.2. Efficacy in Maintaining Cherry Tomato Quality

**Figure 6:**
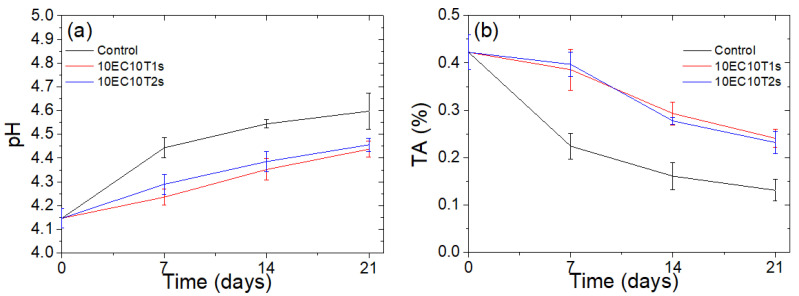
pH (**a**) and titratable acidity (**b**) of cherry tomatoes stored with or without the presence of microparticles.

The efficiency in maintaining the freshness and quality of cherry tomatoes stored at ambient conditions was evaluated for the microparticle formulations with the highest thymol content. During storage, tomato pH is estimated to rise and titratable acidity to decline as organic acids degrade to other sugars due to respiration or due to the action of microorganisms. Therefore, smaller pH or titratable acidity deviations from the initial value indicate enhanced quality characteristics, while in the literature the acceptable tomato pH range is noted to be 4.5 to 5 [61]. As observed in Figure 6a, the control tomatoes indicate an unacceptable pH on day 14, while the pH of the tomatoes stored into the packaging with the microparticles is within the acceptable levels even after 21 days of storage at ambient conditions. Both microparticle formulations managed to maintain a notably more stable pH and titratable acidity (Figure 6b) of the tomatoes compared to the control. This finding is in accordance with the established literature, which denotes the beneficial role of essential oils in suppressing ethylene production [89,90,91], a plant hormone responsible for activating the respiratory enzymes, increasing respiration rates and decomposing carbohydrates into sugars. Overall, the beneficial role of microparticles in maintaining the quality of tomatoes stored under ambient conditions is evident from the results.

## 4. Conclusions

In this study, thymol was entrapped, for the first time, into nontoxic, edible ethyl cellulose microparticles following a green method. Five different microparticle formulations with two different amounts of thymol (5% *w*/*w* and 10% *w*/*w*) were fabricated.

The incorporation of thymol into ethyl cellulose microparticles enhanced the thermal stability of thymol by 10 °C at a thymol to polymer ratio of 1:1, while a further increase in this ratio to 2:1 resulted in an additional enhancement of thermal stability. All microparticles exhibited a gradual release profile of thymol, best characterized by the Higuchi kinetic model. An increased polymer to thymol ratio resulted in a more gradual release of the active compound. The formulations with higher polymer or surfactant content demonstrated slower release, and this related to their hydrophobic nature. Thus, a desired release profile was achieved by tuning the concentration of the polymer or the surfactant in the formulation. The finding is noteworthy, as the proposed microparticles demonstrated a prolonged sustained release exceeding 700 h in contrast with other studies [92,93] that demonstrate substantial release within 8–25 h of interaction with food simulants or up to 250 h of interaction with nonfood simulants [81,83,84,85].

The microparticles with the highest thymol content exhibited significant antimicrobial activity against *E. coli*, which was maintained for at least one month of microparticle storage. To evaluate their effectiveness in preserving food quality, the microparticles were attached on tapes and employed, for the first time, as pads within the packaging of cherry tomatoes. The pH of cherry tomatoes stored without the microparticle pads became unacceptable by day 14, whereas the pH and titratable acidity of tomatoes stored with the thymol microparticle pads remained notably stable for at least 21 days of storage. The findings indicate the advantageous role of thymol microparticles in maintaining the quality characteristics of cherry tomatoes and extending their shelf-life.

Overall, it can be concluded that although higher encapsulation efficiencies may be observed in the literature during encapsulation of phenolic-based substances, the proposed methodology is advantageous for food contact applications since it does not employ any toxic or hazardous substances and results in sustained release of bioactivity and considerable enhancement of the antimicrobial resistance and quality of food during storage for a prolonged period of time. Furthermore, the proposed microcapsules could be incorporated in any type of packaging, and this could result in a facile methodology for the development of active packaging. However, further research is required to enhance the recovery ability of the process through the elimination of agglomeration. This would allow more particles to be sustained during the material’s production, offering higher recovery, which would result in a more viable encapsulation methodology.

## Figures and Tables

**Table 1:** Different formulations of the microparticles with thymol.

Thymol (% *w*/*v*)	Ethyl Cellulose (% *w*/*v*)	Span (% *v*/*v*)	Sample Name
5	5	1	5EC5T1s
5	5	2	5EC5T2s
10	5	2	10EC5T2s
10	10	1	10EC10T1s
10	10	2	10EC10T2s

**Table 2:** Ratio of the peak integral at 1739 cm^−1^ due to Span 80 to the integral of the peak at 806 cm^−1^ due to thymol.

Sample	Span/Thymol ^1^	SD
5EC5T1s (a)	0.44 ^a–c^	±0.02
5EC5T2s (b)	0.62 ^b^	±0.08
10EC5T2s (c)	0.60 ^c–d^	±0.01
10EC10T1s (d)	0.37 ^d–c^	±0.02
10EC10T2s (e)	0.53 ^e^	±0.06

^1^ Letters denote a statistical significance difference among the various formulations (a–e) (*p* < 0.05).

**Table 3:** Thymol content in each microparticle formulation and the temperature at which 20% (*w*/*w*) mass loss of thymol occurs.

Sample	LC%	EE%	T_20_ (°C)
Thymol	-	-	130
5EC5T1S	6.15	12.30	141
5EC5T2S	5.90	11.80	141
10EC5T2S	5.62	11.24	150
10EC10T1S	13.25	26.50	140
10EC10T2S	12.42	24.84	143

**Table 4:** Release kinetic models fitted on the experimental data.

Sample	Zero-Order		First-Order		Hixson– Crowell		Higuchi		Korsmeyer–Peppas	
	R^2^	K_0_	R^2^	K_1_	R^2^	K_HC_	R^2^	K_H_	R^2^	n
5EC5T1s	0.741	0.139	0.422	0.002	0.544	0.004	**0.929**	3.738	**0.902**	0.388
5EC5T2s	0.763	0.143	0.474	0.004	0.584	0.004	**0.947**	3.844	**0.947**	0.390
10EC5T2s	**0.802**	0.149	0.484	0.002	0.614	0.004	**0.968**	3.951	**0.950**	0.452
10EC10T1s	0.736	0.140	0.473	0.003	0.572	0.004	**0.934**	3.788	**0.950**	0.360
10EC10T2s	0.788	0.142	0.477	0.002	0.600	0.004	**0.959**	3.767	**0.935**	0.382

## Data Availability

Data are included within this article and Supplementary Materials.

## Supplementary Materials

The following supporting information can be downloaded at https://www.mdpi.com/article/10.3390/polym16233396/s1: Figure S1: DTG curves of thymol and microparticle formulations with and without thymol; Figure S2: DSC thermographs of thymol and microparticle formulations with and without thymol; Figure S3: Higuchi fitting of the release profile for 5EC5T1s (a), 5EC5T2s (b), 10EC10T1s (c), 10EC5T2s (d) and 10EC10T2s (e) microparticles.
