# Supplementary material for: Encapsulation of Thymol in Ethyl Cellulose-Based Microspheres and Evaluation of Its Sustained Release for Food Applications
Source: Polymers (Basel). 2024 Dec 2;16(23):3396. doi: 10.3390/polym16233396 (PMC11644189; doi:10.3390/polym16233396)
Supplement: Supplementary file 1 [file polymers-16-03396-s001.zip › polymers-3315569-supplementary.pdf]

polymers

MDPI

Article

Encapsulation of Thymol in Ethyl Cellulose-Based Microspheres and Evaluation of Its Sustained Release for Food Applications

Iro Giotopoulou [1], Haralambos Stamatis [2] and Nektaria-Marianthi Barkoula [1,*]

[1] Department of Materials Science and Engineering, University of Ioannina, GR-45110, Ioannina, Greece; i.giotopoulou@uoi.gr
[2] Department of Biological Applications and Technology, University of Ioannina, GR-45110, Ioannina, Greece; hstamati@uoi.gr
* Correspondence: nbarkoul@uoi.gr; Tel.: +30-26510-08003

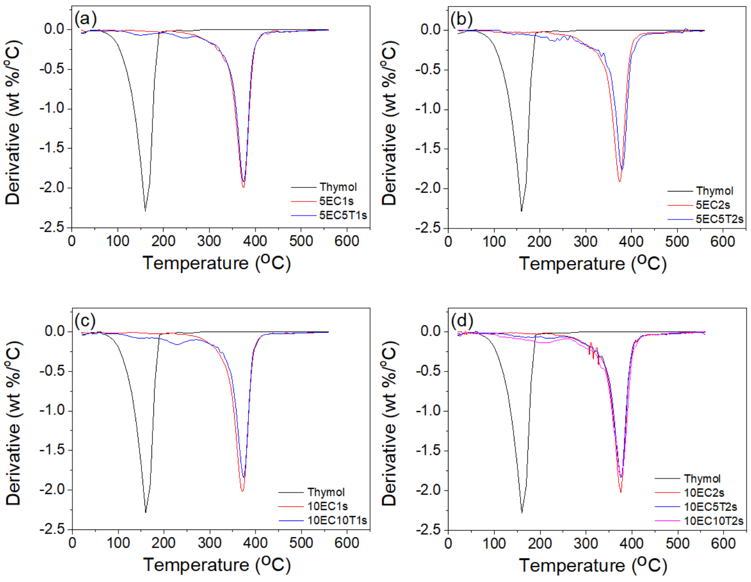

Figure S1: DTG curves of thymol and microparticle formulations with and without thymol.

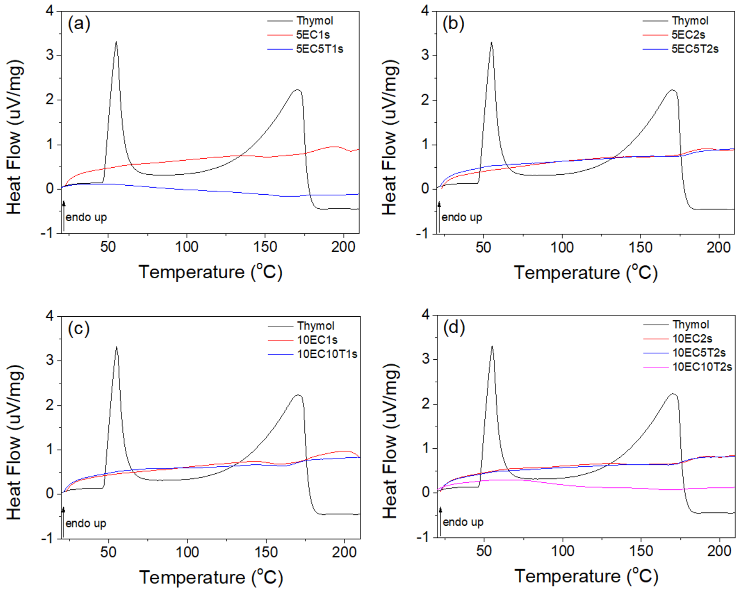

Figure S2: DSC thermographs of thymol and microparticle formulations with and without thymol.

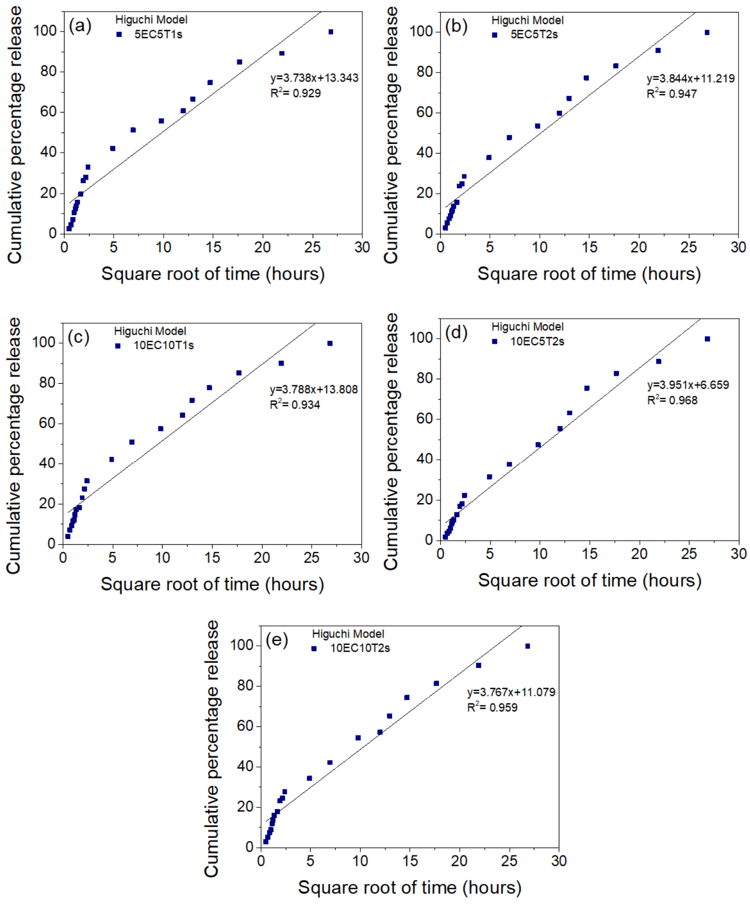

Figure S3. Higuchi fitting of the release profile for 5EC5T1s (a), 5EC5T2s (b), 10EC10T1s (c), 10EC5T2s (d), and 10EC10T2s (e).
